# Fixed-Time Leader-Following Consensus Tracking Control for Nonliear Multi-Agent Systems under Jointly Connected Graph

**DOI:** 10.3390/e24081130

**Published:** 2022-08-15

**Authors:** Meng Zhao, Chan Gu, Le Zhao, Yungang Liu

**Affiliations:** 1 School of Electrical and Control Engineering, Shaanxi University of Science and Technology, Xi’an 720021, China; 2 School of Control Science and Engineering, Shandong University, Jinan 250100, China

**Keywords:** multi-agent systems, fixed-time consensus, jointly connected graph, nonlinear, disturbance

## Abstract

This paper researches the fixed-time leader-following consensus problem for nonlinear multi-agent systems (MASs) affected by unknown disturbances under the jointly connected graph. In order to achieve control goal, this paper designs a fixed-time consensus protocol, which can offset the unknown disturbances and the nonlinear item under the jointly connected graph, simultaneously. In this paper, the states of multiple followers can converge to the state of the leader within a fixed time regardless of the initial conditions rather than just converging to a small neighborhood near the leader state. Finally, a simulation example is given to illustrate the theoretical result.

## 1. Introduction

Over the years, multi-agent systems have been widely considered in many fields because of their advantages of low cost and high efficiency [1,2,3,4]. As everyone knows, the consensus problem is a vital one in the field of cooperative control of multi-agent systems, which is the basis for the study of other cooperative control problems.

In the study of consensus problems, convergence rate is often regarded as an important performance index to measure the excellence of the designed control protocol. Therefore, in terms of convergence rate, the consensus problem of MASs can be divided into the asymptotic consensus, the finite time consensus and the fixed-time consensus generally. Firstly, the asymptotic consensus problem can be achieved when time approaches infinity [5,6]. However, in practical application, it is often expected that each agent can reach consensus within a limited time. Then, the finite time consensus comes into being. Compared with the asymptotic consensus, the convergence speed of the finite time consensus is obviously faster, which possesses stronger robustness and higher control precision [7,8,9,10,11,12,13]. However, the finite time consensus still has obvious limitations; that is, its convergence time is related to the initial values. In order to solve the limitation of finite time consensus of MASs, Polyakov first proposed the concept of the fixed-time stability in 2012 [14]. On the basis of retaining the advantages of the finite time consensus, the convergence time of multi-agent systems is independent of the initial value.

Furthermore, due to its obvious advantages, the research of fixed-time consensus has developed rapidly in recent years. Firstly, work [15] studied the fixed-time consensus problem for simple second-order integrator multi-agent systems. Moreover, work [16] studied the second-order system with disturbances whose upper bounds were known, and it designed an observer-based distributed fixed-time consensus protocol. Moreover, work [17,18,19] researched the fixed-time consensus problem of first-order nonlinear systems. Among them, work [18] studied the fixed-time consensus problem of nonlinear multi-agent systems subjected to external disturbances and employed adaptive methods to solve the external unknown disturbances and nonlinear problems. In addition, work [20] proposed an adaptive protocol based on high-order observer, which is applied to study the fixed-time leader-following consensus of high-order nonlinear systems, where the nonlinear term satisfied the Lipschitz condition and the Lipschitz constant was known. All of the above are studied under the fixed graph, and there are many studies on the fixed-time consensus under the switching graph. In 2018, work [21] studied the double integrator system under a jointly connection graph, and they adopted distributed protocol to make MASs achieve fixed-time consensus. Furthermore, work [22] studied the problem of fixed-time random consensus of multi-agent systems and designed a series of non-Lipschitz protocol under fixed topology and switching topology, respectively. In addition, work [23] proposed a backstepping distributed control model to design a fixed-time state observer, which could solve the formation problem of multiple UAVs. On the basis of the backstepping method, work [24] introduced a neural network and designed a novel fixed-time adaptive protocol to solve the fixed-time consensus problem of nonlinear multi-agent systems under switching graph. In addition, work [25] uses fuzzy logic control to make higher-order systems achieve practical consensus in a fixed time. However, if a deep learned recurrent type-3 fuzzy system is further combined, the uncertainty modeling of nonlinear systems can be better solved on the basis of the above papers, as mentioned in [26].

Overall, the research of the fixed-time consensus problem needs further improvement. In terms of the dynamics of MASs, many existing achievements do not consider the nonlinear multi-agent systems with disturbances [14,17,20,27,28,29], which is relatively limited. In terms of the communication graphs, most of the studies in the literature related with the fixed-time consensus focused on fixed graphs, while there is not enough research on switching topology [21,22,30].

Inspired by the literature above, this paper studies the fixed-time leader-following consensus of nonlinear multi-agent systems for a jointly connected graph, which is a more difficult system than the one used in [17,18,29]. Then, since the jointly connected graph is not always connected, a novel fixed-time consensus protocol based on a pointed assumption is designed, which can solve both nonlinear terms and unknown disturbances. In this paper, the states of multiple followers can converge to the state of the leader within a fixed time regardless of the initial conditions rather than just converging to a small neighborhood near the leader state. Eventually, the feasibility of the fixed-time consensus protocol is proved strictly by using Lyapunov stability lemma and classical matrix theory.

The remaining sections of this paper are divided as follows. Some important lemmas and the basic algebraic graph theory used in this paper are introduced in Section 2. Section 3 is dedicated to describe the main results of this paper, which consists of three sections, namely problem formulation, the design of the fixed-time consensus protocol, and the corresponding stability analysis. Section 4 uses MATLAB for simulation verification. The conclusion is given in Section 5.

## 2. Preliminaries

### 2.1. Notations

Notations R, $R_{+}$, $R^{n}$ and $R^{n\times n}$ represent the real number set, positive real number set, *n*-dimensional real vector space and n×n matrix, respectively. Then, the symbol 1 is the column vector of n×1 with all elements 1. $I_{n}$ is the *n*-dimensional identity matrix. Define $x={\left[x_{1},x_{2},\cdots ,x_{n}\right]}^{T}\in R^{n}$, i=1,⋯,n, $x^{r}={\left[{x_{1}}^{r},\cdots ,{x_{n}}^{r}\right]}^{T}$, r∈R, and $sign\left(x\right)={\left[sign\left(x_{1}\right),sign\left(x_{2}\right),\cdots ,sign\left(x_{n}\right)\right]}^{T}$, where sign(·) is sign function; that is,

$$\begin{array}{c} sign\left(\phi \right)=\left\{\begin{array}{cc} \; & 1,\phi >0\\ \; & 0,\phi =0\\ \; & -1,\phi <0. \end{array}\right. \end{array}$$

The main notations used in this article are shown in Table 1 below.

### 2.2. Definition and Lemmas

For the convenience of the following description, this section makes unified definitions.

**Definition** **1.** *For $\forall x=\left[x_{1},\cdots ,x_{n}\right]\in R^{n}$, p-norm is defined as*

$$\begin{array}{c} {∥x∥}_{p}^{p}=|x_{1}{|}^{p}+|x_{2}{|}^{p}+\cdots +{\left|x_{n}\right|}^{p}, \end{array}$$

*where p>0.*

The following lemmas are required in this paper. In the meanwhile, they play a crucial role in analyzing the fixed-time consensus of MASs.

Consider the following nonlinear system

(1) $$\begin{array}{c} \dot{x}\left(t\right)=f\left(t,x\right),x\left(0\right)=x_{0}, \end{array}$$

where $x\in R^{n}$, $f\left(\cdot \right):R_{+}\times R^{n}\to R^{n}$ is a nonlinear function. The solution of (1) can be understood in terms of Filippov if f(t,x) is not continuous. Assume that the origin is an equilibrium point of (1).

**Lemma** **1** ([14,31])**.** *If there exists a continuous radial unbounded positive definite function V(x), such that $\dot{V}\left(x\right)\leq -K_{1}V^{p}\left(x\right)-K_{2}V^{q}\left(x\right)$, where constant $K_{1}$, $K_{2}>0$, p>1, 0<q<1, then the origin of system (1) is globally fixed-time stable, where the settling time function T could be estimated as $T\leq T_{max}:=\frac{1}{K_{1}\left(p-1\right)}+\frac{1}{K_{2}\left(1-q\right)}$. Furthermore, if $p=1+\frac{1}{\mu }$, $q=1-\frac{1}{\mu }$, where μ>1, then the upper bound of convergence time is represented as $T_{max}:=\frac{\pi \mu }{2\sqrt{K_{1}K_{2}}}$.*

**Lemma** **2** ([32])**.** *For any vector $x\in R^{n}$, the following inequality holds*

$$\begin{array}{c} {∥x∥}_{p}\leq {∥x∥}_{r}\leq n^{\frac{1}{r}-\frac{1}{p}}{∥x∥}_{p}. \end{array}$$

*where 0<r<p.*

**Lemma** **3** ([32])**.** *For any $x\in R_{+}$, $y\in R_{+}$, then*

$$\begin{array}{c} \sqrt{xy}\leq \frac{x+y}{2}. \end{array}$$

**Lemma** **4** ([33])**.** *For a directed graph, if there is a directed spanning tree whose root is a leader, the Laplacian matrix L associated with the directed graph has only one eigenvalue of 0, the other eigenvalues are positive, and the eigenvector of 0 eigenvalue is 1.*

### 2.3. Algebraic Graph Theory

A graph is represented by G=(V,E,A), where $V=\left\{v_{1},v_{2},\cdots ,v_{n}\right\}$ is a node set, E⊆V×V is an edge set, and A is the adjacency matrix. If $(v_{j},v_{i})\in E$, then the agent $v_{j}$ can obtain information from the agent $v_{i}$. For an edge $\left(v_{j},v_{i}\right)$, node $v_{i}$ is called the parent node of $v_{j}$, $v_{j}$ is called the child node of $v_{i}$, and $v_{i}$ is a neighbor of $v_{j}$. An adjacency matrix associated with the graph G is defined as $A=\left[a_{ij}\right]\in R^{n\times n}$, where $a_{ij}>0$, when $(v_{j},v_{i})\in E$; $a_{ij}=0$, otherwise. Note that $a_{ij}$ represents the weight for the edge $\left(v_{j},v_{i}\right)$. The Laplacian matrix is defined as $L=\left[L_{ij}\right]\in R^{n\times n}$, where $L_{ii}=\sum_{i\neq j}a_{ij}$ and $L_{ij}=-a_{ij}$, i≠j. In addition, the Laplacian matrix is expressed as L=D−A, where D= diag$\left\{d_{1},d_{2},\cdots ,d_{n}\right\}$ is a degree matrix with $d_{i}=\sum_{j=1}^{n}a_{ij}$.

In addition, a directed graph is called a strong connected graph if any node has a directed path to other nodes. It is worth noting that a connected graph is the premise of studying the consensus problem. For a directed graph, if a node $v_{i}$ can reach any other node through a directed path, the communication topology is said to have a directed spanning tree with $v_{i}$ as the root node.

A switching graph can be described by $G_{\sigma (t)}=\left(V^{\sigma (t)},E^{\sigma (t)},A^{\sigma (t)}\right)$, where σ(t): R→P and P is a finite set. The communication graph proposed in this paper is a switching graph with jointly connectivity, that is, consider a series of infinite sequences consisting of continuous time intervals $\left[t_{i},t_{i+1}\right)$, i=0,1,⋯,n, where $t_{0}=0$, $t_{i+1}-t_{i}\leq T$, and *T* is a positive constant, while let $N_{i}^{\sigma (t)}$ represent the neighbor set of the *i*-th agent at different time intervals. Then, each interval $\left[t_{i},t_{i+1}\right)$ can consist of an integer $p_{i}$ continuous sub-time intervals $\left[t_{i}^{0},t_{i}^{1}\right),\cdots ,\left[t_{i}^{j},t_{i}^{j+1}\right),\cdots ,\left[t_{i}^{p_{1}-1},t_{i}^{p_{i}}\right)$, where $t_{i}^{0}=t_{i}$, $t_{i}^{p_{i}}=t_{i+1}$, $t_{i}^{j+1}-t_{i}^{j}\geq S$, and *S* is a positive constant. The Laplacian matrix $L_{\sigma (t)}$ associated with the jointly connected graph $G_{\sigma (t)}$ is represented by

$$\begin{array}{c} L^{\sigma (t)}=\left[\begin{array}{cc} 0 & 0_{n\times 1}\\ L_{2}^{\sigma (t)} & L_{1}^{\sigma (t)} \end{array}\right]. \end{array}$$

## 3. Main Results

### 3.1. Problem Formulation

Consider that the system contains *n* + 1 agents, numbered 0,⋯,n, respectively, in which agent 0 is the leader and the other agents are followers. The dynamics of the leader is described by

(2) $$\begin{array}{c} {\dot{x}}_{0}\left(t\right)=u_{0}+f\left(t,x_{0}\right). \end{array}$$

The dynamics of the *i*-th agent is represented by

(3) $$\begin{array}{c} {\dot{x}}_{i}\left(t\right)=u_{i}+f\left(t,x_{i}\right)+d_{i}\left(t\right),i=1,\cdots ,n, \end{array}$$

where $x_{0}\in R^{n}$, $u_{0}\in R^{n}$, $x_{i}\in R^{n}$ and $u_{i}\in R^{n}$ represent the state of a leader, the control input of a leader, the state of the *i*-th follower and the control input of the *i*-th follower, $d_{i}\left(t\right)\in R^{n}$ represents the uncertain disturbances, $f(t,x_{0})$ and $f(t,x_{i})$: $R_{+}\times R^{n}\to R^{n}$ is the continuous nonlinear function. Without losing generality, this paper defaults that the leader cannot receive information from the followers, and only part of the followers can receive the state information of the leader.

**Assumption** **1.** *The disturbance $d_{i}\left(t\right)$ of each agent is continuously differentiable and uniformly bounded, i.e., $d_{i}\left(t\right)\leq {∥d_{i}\left(t\right)∥}_{\infty }$.*

**Assumption** **2.** *The leader has a non-zero control input $u_{0}$, and $u_{0}$ has the known upper bound, i.e., $u_{0}\leq {∥u_{0}∥}_{\infty }$.*

**Assumption** **3.** *For any $x_{i}$, $x_{j}\in R^{n}$, there exists known positive constant θ, such that*

$$\begin{array}{c} ∥f\left(t,x_{i}\right)-f\left(t,x_{j}\right)∥\leq \theta ∥x_{i}-x_{j}∥. \end{array}$$

**Assumption** **4.** *Consider a series of infinite sequences consisting of continuous time intervals $\left[t_{i},t_{i+1}\right)$, i=0,1,⋯,n, where $t_{0}=0$, $t_{i+1}-t_{i}\leq T$, and T is a positive constant, while $N_{i}^{\sigma (t)}$ represents the neighbor set of the* i*-th agent at different time intervals. Furthermore, the entire interval $\left[t_{i},t_{i+1}\right)$ can consist of an integer $p_{i}$ contiguous sub-time intervals $\left[t_{i}^{0},t_{i}^{1}\right),\cdots ,\left[t_{i}^{j},t_{i}^{j+1}\right),\cdots ,\left[t_{i}^{p_{i}-1},t_{i}^{p_{i}}\right)$, where $t_{i}^{0}=t_{i}$, $t_{i}^{p_{i}}=t_{i+1}$, $t_{i}^{j+1}-t_{i}^{j}\geq S$, and S is a positive constant. Moreover, the subgraph does not need to have a directed spanning tree with the leader as the root node at each sub-time interval, the jointly connected graph $G_{\sigma (t)}={⋃}_{j=0}^{p_{i}-1}G_{i}^{j}$ contains a directed tree in each time interval $\left[t_{i},t_{i+1}\right)$, and a leader is a root node.*

The control objective of this paper is to design a control protocol $u_{i}$ such that *n* followers (3) can converge to the leader state (2) in a finite time under the jointly connected graph, and the convergence time is independent of the initial state of the system; that is, for any initial value $x_{i}\left(0\right)$, there exists a fixed time $T_{max}$, such that

$$\begin{array}{c} \lim_{t\to T_{max}}{∥x_{i}\left(t\right)-x_{0}\left(t\right)∥}_{2}=0,\forall t>T_{max}. \end{array}$$

In order to achieve the above control objective, the aforementioned assumptions should be satisfied.

### 3.2. Fixed-Time Consensus Protocol

As mentioned above, the aim of this paper is to study the fixed-time consensus for systems (2) and (3). Therefore, in each time interval $\left[t_{i},t_{i+1}\right)$, the control protocol for each follower is designed

(4) $$\begin{array}{cc} u_{i}\left(t\right)=\; & -\alpha {\left[\sum_{j=1}^{n}a_{ij}\left(x_{i}-x_{j}\right)+a_{i0}\left(x_{i}-x_{0}\right)\right]}^{\left(1-b\right)}\\ \; & -\beta {\left[\sum_{j=1}^{n}a_{ij}\left(x_{i}-x_{j}\right)+a_{i0}\left(x_{i}-x_{0}\right)\right]}^{\left(1+b\right)}\\ \; & -\gamma sign\left(\sum_{j=1}^{n}a_{ij}\left(x_{i}-x_{j}\right)+a_{i0}\left(x_{i}-x_{0}\right)\right), \end{array}$$

where α, β, γ>0, 0<b<1, and $b=\frac{2q}{2q+1}$, q=1,⋯,n. Then, the first two terms of (4) are dedicated to solve nonlinear terms and ensure that the systems (2) and (3) achieve the fixed-time consensus, while the last term is employed to eliminate unknown disturbances.

Let $e_{i}\left(t\right)=x_{i}\left(t\right)-x_{0}\left(t\right)$, i=1,⋯,n. By substituting (4) into systems (2) and (3), the dynamics of the error system can be obtained

(5) $$\begin{array}{cc} {\dot{e}}_{i}\left(t\right)=\; & -\alpha {\left(\sum_{j=1}^{n}a_{ij}\left(e_{i}\left(t\right)-e_{j}\left(t\right)\right)+a_{i0}^{\sigma (t)}e_{i}\left(t\right)\right)}^{1-b}\\ \; & -\beta {\left(\sum_{j=1}^{n}a_{ij}^{\sigma (t)}\left(e_{i}\left(t\right)-e_{j}\left(t\right)\right)+a_{i0}e_{i}\left(t\right)\right)}^{1+b}\\ \; & -\gamma sign\left(\sum_{j=1}^{n}a_{ij}\left(e_{i}\left(t\right)-e_{j}\left(t\right)\right)+a_{i0}e_{i}\left(t\right)\right)\\ \; & +d_{i}\left(t\right)+f\left(t,x_{i}\right)-f\left(t,x_{0}\right)-u_{0}, \end{array}$$

Let $E={\left[e_{1}\left(t\right),\cdots ,e_{n}\left(t\right)\right]}^{T}$. We can obtain the compact form of (5) as follows

(6) $$\begin{array}{cc} \dot{E}=\; & -\alpha {\left((L_{1}^{\sigma (t)}⊗I_{n})E\right)}^{1-b}-\beta {\left((L_{1}^{\sigma (t)}⊗I_{n})E\right)}^{1+b}\\ \; & -\gamma sign\left((L_{1}^{\sigma (t)}⊗I_{n})E\right)-1⊗u_{0}\\ \; & +F\left(t,x\right)-F\left(t,x_{0}\right)+D, \end{array}$$

where $F\left(t,x\right)={\left[f\left(t,x_{1}\right),\cdots ,f\left(t,x_{n}\right)\right]}^{T}$, $F\left(t,x_{0}\right)={\left[f\left(t,x_{0}\right),\cdots ,f\left(t,x_{0}\right)\right]}^{T}$, $D=[d_{1}\left(t\right),\cdots ,$$d_{n}{\left(t\right)]}^{T}$. According to Lemma 4, $L_{1}^{\sigma (t)}$ is a positive define matrix.

**Theorem** **1.** *Under Assumptions 1–4, the multi-agent systems (2) and (3) can achieve the fixed-time consensus under the control protocol (4), and the settling time T can be estimated as*

(7) $$\begin{array}{c} T=\frac{1}{\bar{\alpha }{\left(\frac{1}{2}\lambda_{min}\left({\left(L_{1}^{\sigma (t)}\right)}^{-1}\right)\right)}^{\frac{b-2}{2}}\cdot \frac{b}{2}}+\frac{1}{\bar{\beta }{\left(\frac{1}{2}\lambda_{min}\left({\left(L_{1}^{\sigma (t)}\right)}^{-1}\right)\right)}^{\frac{-b-2}{2}}\cdot \frac{b}{2}}, \end{array}$$

*where $\bar{\alpha }=\alpha -\frac{1}{2}n\theta {∥\left({\left(L_{1}^{\sigma (t)}\right)}^{-1}⊗I_{d}\right)∥}_{2}$, $\bar{\beta }=\beta n^{-\frac{b}{2}}-\frac{1}{2}n\theta {∥\left({\left(L_{1}^{\sigma (t)}\right)}^{-1}⊗I_{d}\right)∥}_{2}$.*

**Proof.**  Consider the following Lyapunov function candidate

(8) $$\begin{array}{c} V\left(E\right)=\frac{1}{2}E^{T}\left(L_{1}^{\sigma (t)}⊗I_{n}\right)E, \end{array}$$

Since $L_{1}^{\sigma (t)}$ is a positive define matrix, i.e., $L_{1}^{\sigma (t)}>0$, thus, V(E) is positive definite and continuously differentiable. Clearly, the derivative of (8) is shown below

(9) $$\begin{array}{c} \dot{V}\left(E\right)=E^{T}\left(L_{1}^{\sigma (t)}⊗I_{n}\right)\dot{E}. \end{array}$$

Substituting (6) into (9), we have

(10) $$\begin{array}{cc} \dot{V}\left(E\right)=\; & -\alpha E^{T}\left(L_{1}^{\sigma (t)}⊗I_{n}\right){\left(\left(L_{1}^{\sigma (t)}⊗I_{n}\right)E\right)}^{1-b}\\ \; & -\beta E^{T}\left(L_{1}^{\sigma (t)}⊗I_{n}\right){\left(\left(L_{1}^{\sigma (t)}⊗I_{n}\right)E\right)}^{1+b}\\ \; & -\gamma E^{T}\left(L_{1}^{\sigma (t)}⊗I_{n}\right)sign\left(\left(L_{1}^{\sigma (t)}⊗I_{n}\right)E\right)\\ \; & +E^{T}\left(L_{1}^{\sigma (t)}⊗I_{n}\right)\left[F\left(t,x\right)-F\left(t,x_{0}\right)\right]\\ \; & -E^{T}\left(L_{1}^{\sigma (t)}⊗I_{n}\right)\left(1⊗u_{0}\right)+E^{T}\left(L_{1}^{\sigma (t)}⊗I_{n}\right)D. \end{array}$$

Combining the above (10) with Definition 1, the following inequality is obtained

(11) $$\begin{array}{cc} \dot{V}\left(E\right)=\; & -\alpha ∥\left(\left(L_{1}^{\sigma (t)}⊗I_{n}\right)E\right){∥}_{2-b}^{2-b}\\ \; & -\beta ∥\left(\left(L_{1}^{\sigma (t)}⊗I_{n}\right)E\right){∥}_{2+b}^{2+b}\\ \; & -\gamma E^{T}\left(L_{1}^{\sigma (t)}⊗I_{n}\right)sign\left(\left(L_{1}^{\sigma (t)}⊗I_{n}\right)E^{T}\right)\\ \; & +E^{T}\left(L_{1}^{\sigma (t)}⊗I_{n}\right)\left[F\left(t,x\right)-F\left(t,x_{0}\right)\right]\\ \; & -E^{T}\left(L_{1}^{\sigma (t)}⊗I_{n}\right)\left(1⊗u_{0}\right)+E^{T}\left(L_{1}^{\sigma (t)}⊗I_{n}\right)D. \end{array}$$

By using assumptions, it follows from (11) that

(12) $$\begin{array}{cc} \dot{V}\left(E\right)\leq \; & -\alpha ∥\left(\left(L_{1}^{\sigma (t)}⊗I_{n}\right)E\right){∥}_{2-b}^{2-b}\\ \; & -\beta ∥\left(\left(L_{1}^{\sigma (t)}⊗I_{n}\right)E\right){∥}_{2+b}^{2+b}\\ \; & +E^{T}\left(L_{1}^{\sigma (t)}⊗I_{n}\right)\left[F\left(t,x\right)-F\left(t,x_{0}\right)\right]\\ \; & -(\gamma -∥\left(1⊗u_{0}\right){∥}_{\infty }-∥d_{i}\left(t\right){∥}_{\infty })∥\left(L_{1}^{\sigma (t)}⊗I_{n}\right){E∥}_{1}. \end{array}$$

Through selecting sufficiently large γ, such that $\gamma \geq ∥\left(1⊗u_{0}\right){∥}_{\infty }+{∥d_{i}\left(t\right)∥}_{\infty }$, (12) can transform into

(13) $$\begin{array}{cc} \dot{V}\left(E\right)\leq \; & -\alpha ∥\left(\left(L_{1}^{\sigma (t)}⊗I_{n}\right)E\right){∥}_{2-b}^{2-b}\\ \; & -\beta ∥\left(\left(L_{1}^{\sigma (t)}⊗I_{n}\right)E\right){∥}_{2+b}^{2+b}\\ \; & +E^{T}\left(L_{1}^{\sigma (t)}⊗I_{n}\right)\left[F\left(t,x\right)-F\left(t,x_{0}\right)\right], \end{array}$$

where the nonlinear term $E^{T}\left(L_{1}^{\sigma (t)}⊗I_{n}\right)\left[F\left(t,x\right)-F\left(t,x_{0}\right)\right]$ can be rewritten by Assumption 3 and Lemma 2

(14) $$\begin{array}{cc} \; & E^{T}\left(L_{1}^{\sigma (t)}⊗I_{n}\right)\left[F\left(t,x\right)-F\left(t,x_{0}\right)\right]\\ \leq & ∥\left(L_{1}^{\sigma (t)}⊗I_{d}\right)E∥∥F\left(t,x\right)-F\left(t,x_{0}\right)∥\\ \leq & n\theta ∥\left(L_{1}^{\sigma (t)}⊗I_{n}\right){E∥}_{2}{∥E∥}_{2}. \end{array}$$

Let $\xi ={\left[\xi_{1},\cdots ,\xi_{n}\right]}^{T}=\left(L_{1}^{\sigma (t)}⊗I_{d}\right)E$; thus, (14) can turn into

(15) $$\begin{array}{cc} \; & n\theta ∥\left(L_{1}^{\sigma (t)}⊗I_{n}\right){E∥}_{2}{∥E∥}_{2}\\ \leq & n\theta ∥\left({\left(L_{1}^{\sigma (t)}\right)}^{-1}⊗I_{n}\right){∥}_{2}{∥\xi ∥}_{2}^{2}. \end{array}$$

Combining Lemma 3, (15) can be written as

(16) $$\begin{array}{cc} \; & n\theta ∥\left({\left(L_{1}^{\sigma (t)}\right)}^{-1}⊗I_{n}\right){∥}_{2}{∥\xi ∥}_{2}^{2}\\ \leq & n\theta ∥\left({\left(L_{1}^{\sigma (t)}\right)}^{-1}⊗I_{n}\right){∥}_{2}\frac{{∥\xi ∥}_{2}^{2-b}+{∥\xi ∥}_{2}^{2+b}}{2}. \end{array}$$

Moreover, (13) can further change

(17) $$\begin{array}{cc} \dot{V}\left(\xi \right)\leq \; & -{\alpha ∥\xi ∥}_{2-b}^{2-b}-\beta {∥\xi ∥}_{2+b}^{2+b}\\ \; & +{\left(\xi \right)}^{T}\left[F\left(t,x\right)-F\left(t,x_{0}\right)\right]. \end{array}$$

Furthermore, the following inequality can be obtained by substituting (16) into (17)

(18) $$\begin{array}{cc} \dot{V}\left(\xi \right)\leq \; & -{\alpha ∥\xi ∥}_{2-b}^{2-b}-\beta {∥\xi ∥}_{2+b}^{2+b}\\ \; & +\frac{1}{2}n\theta ∥\left({\left(L_{1}^{\sigma (t)}\right)}^{-1}⊗I_{n}\right){∥}_{2}{∥\xi ∥}_{2}^{2-b}\\ \; & +\frac{1}{2}n\theta ∥\left({\left(L_{1}^{\sigma (t)}\right)}^{-1}⊗I_{n}\right){∥}_{2}{∥\xi ∥}_{2}^{2+b}. \end{array}$$

According to 2−b<2 and 2+b>2, and Lemma 2 gives us that

(19) $$\begin{array}{cc} \; & {∥\xi ∥}_{2-b}\geq {∥\xi ∥}_{2}, \end{array}$$

(20) $$\begin{array}{cc} \; & {∥\xi ∥}_{2+b}\geq n^{\frac{1}{2+b}-\frac{1}{2}}{∥\xi ∥}_{2}. \end{array}$$

Therefore,

(21) $$\begin{array}{cc} \; & {∥\xi ∥}_{2-b}^{2-b}\geq {∥\xi ∥}_{2}^{2-b}, \end{array}$$

(22) $$\begin{array}{cc} \; & {∥\xi ∥}_{2+b}^{2+b}\geq n^{1-\frac{2+b}{2}}{∥\xi ∥}_{2}^{2+b}. \end{array}$$

Then, (18) can be further changed from (21) and (22) above

(23) $$\begin{array}{cc} \dot{V}\left(\xi \right)\leq \; & -{\alpha ∥\xi ∥}_{2}^{2-b}-\beta n^{1-\frac{2+b}{2}}{∥\xi ∥}_{2}^{2+b}\\ \; & +\frac{1}{2}n\theta ∥\left({\left(L_{1}^{\sigma (t)}\right)}^{-1}⊗I_{n}\right){∥}_{2}{∥\xi ∥}_{2}^{2-b}\\ \; & +\frac{1}{2}n\theta ∥\left({\left(L_{1}^{\sigma (t)}\right)}^{-1}⊗I_{n}\right){∥}_{2}{∥\xi ∥}_{2}^{2+b}\\ = & -\left(\alpha -\frac{1}{2}n\theta {∥\left({\left(L_{1}^{\sigma (t)}\right)}^{-1}⊗I_{n}\right)∥}_{2}\right){∥\xi ∥}_{2}^{2-b}\\ \; & -\left(\beta n^{-\frac{b}{2}}-\frac{1}{2}n\theta {∥\left({\left(L_{1}^{\sigma (t)}\right)}^{-1}⊗I_{n}\right)∥}_{2}\right){∥\xi ∥}_{2}^{2+b}. \end{array}$$

Selecting suitable α and β, such that $\alpha -\frac{1}{2}n\theta {∥\left({\left(L_{1}^{\sigma (t)}\right)}^{-1}⊗I_{n}\right)∥}_{2}>0$ and $\beta n^{-\frac{b}{2}}-\frac{1}{2}n\theta ∥$$\left({\left(L_{1}^{\sigma (t)}\right)}^{-1}⊗I_{n}\right){∥}_{2}>0$, then

(24) $$\begin{array}{c} \dot{V}\left(\xi \right)\leq -\bar{\alpha }{∥\xi ∥}_{2}^{2-b}-\bar{\beta }{∥\xi ∥}_{2}^{2+b}, \end{array}$$

where $\bar{\alpha }=\alpha -\frac{1}{2}n\theta {∥\left({\left(L_{1}^{\sigma (t)}\right)}^{-1}⊗I_{n}\right)∥}_{2}$, $\bar{\beta }=\beta n^{-\frac{b}{2}}-\frac{1}{2}n\theta {∥\left({\left(L_{1}^{\sigma (t)}\right)}^{-1}⊗I_{n}\right)∥}_{2}$.

Moreover, (8) can be rewritten as

(25) $$\begin{array}{cc} V(\xi )=\; & \frac{1}{2}\xi^{T}\left({\left(L_{1}^{\sigma (t)}\right)}^{-1}⊗I_{n}\right)\xi \\ \leq & \frac{1}{2}\lambda_{min}\left({\left(L_{1}^{\sigma (t)}\right)}^{-1}\right){∥\xi ∥}_{2}^{2}. \end{array}$$

Thus, (24) can be given a new expression as follows

(26) $$\begin{array}{cc} \dot{V}\left(\xi \right)\leq \; & -\bar{\alpha }{∥\xi ∥}_{2}^{2-b}-\beta {∥\xi ∥}_{2}^{2+b}\\ = & -\bar{\alpha }{\left[\frac{1}{2}\lambda_{min}\left({\left(L_{1}^{\sigma (t)}\right)}^{-1}\right)\right]}^{\frac{b-2}{2}}(\left(\frac{1}{2}\lambda_{min}{\left(L_{1}^{\sigma (t)}\right)}^{-1}\right){∥\xi ∥}_{2}^{2}{)}^{\frac{2-b}{2}}\\ \; & -\bar{\beta }{\left[\frac{1}{2}\lambda_{min}\left({\left(L_{1}^{\sigma (t)}\right)}^{-1}\right)\right]}^{\frac{-b-2}{2}}(\left(\frac{1}{2}\lambda_{min}{\left(L_{1}^{\sigma (t)}\right)}^{-1}\right){∥\xi ∥}_{2}^{2}{)}^{\frac{2+b}{2}}\\ = & -\bar{\alpha }{\left[\frac{1}{2}\lambda_{min}\left({\left(L_{1}^{\sigma (t)}\right)}^{-1}\right)\right]}^{\frac{b-2}{2}}{\left(V\left(\xi \right)\right)}^{\frac{2-b}{2}}\\ \; & -\bar{\beta }{\left[\frac{1}{2}\lambda_{min}\left({\left(L_{1}^{\sigma (t)}\right)}^{-1}\right)\right]}^{\frac{-b-2}{2}}{\left(V\left(\xi \right)\right)}^{\frac{2+b}{2}}. \end{array}$$

Then, let $K_{1}=\bar{\alpha }{\left(\frac{1}{2}\lambda_{min}\left({\left(L_{1}^{\sigma (t)}\right)}^{-1}\right)\right)}^{\frac{b-2}{2}}$ and $K_{2}=\bar{\beta }{\left(\frac{1}{2}\lambda_{min}\left({\left(L_{1}^{\sigma (t)}\right)}^{-1}\right)\right)}^{\frac{-b-2}{2}}$; thus, (26) can be shown as follows

(27) $$\begin{array}{c} \dot{V}\left(\xi \right)\leq -K_{1}V{\left(\xi \right)}^{\frac{2-b}{2}}-K_{2}V{\left(\xi \right)}^{\frac{2+b}{2}}. \end{array}$$

From (27), $\dot{V}\left(\xi \right)\leq 0$; thus, $\dot{V}\left(\xi \right)$ is a decreasing function. Therefore, there exists $\lim_{t\to \infty }V\left(\xi \right)$, that is, V(ξ) is bounded. While ${\left(L_{1}^{\sigma (t)}\right)}^{-1}$ and $I_{n}$ is bounded in (27), thus, ξ is also bounded. For $\xi =(L_{1}^{\sigma (t)}⊗I_{n})E$, where $L_{1}^{\sigma (t)}$ and $I_{n}$ is bounded, thus, E is also bounded. In addition, since $u_{0}$ is bounded, $\dot{E}$ is bounded by combining (6), thus $\dot{\xi }$ is also bounded. Conclusively, since the relative information between agents is bounded, the control protocol $u_{i}\left(t\right)$ is bounded.

Overall, using the above definition, we can obtain that $K_{1}$, $K_{2}>0$, $\frac{2-b}{2}$ and $\frac{2+b}{2}$ are all even power. Combining (27) and Lemma 1, the fixed-time consensus problem of (2) and (3) is solved, and the estimated value of the settling time is

(28) $$\begin{array}{cc} T\leq T_{max}:=\; & \frac{1}{\bar{\alpha }{\left[\frac{1}{2}\lambda_{min}\left({\left(L_{1}\right)}^{-1}\right)\right]}^{\frac{b-2}{2}}\cdot \frac{b}{2}}\\ \; & +\frac{1}{\bar{\beta }{\left[\frac{1}{2}\lambda_{min}\left({\left(L_{1}\right)}^{-1}\right)\right]}^{\frac{-b-2}{2}}\cdot \frac{b}{2}}. \end{array}$$

□

**Figure 1:**
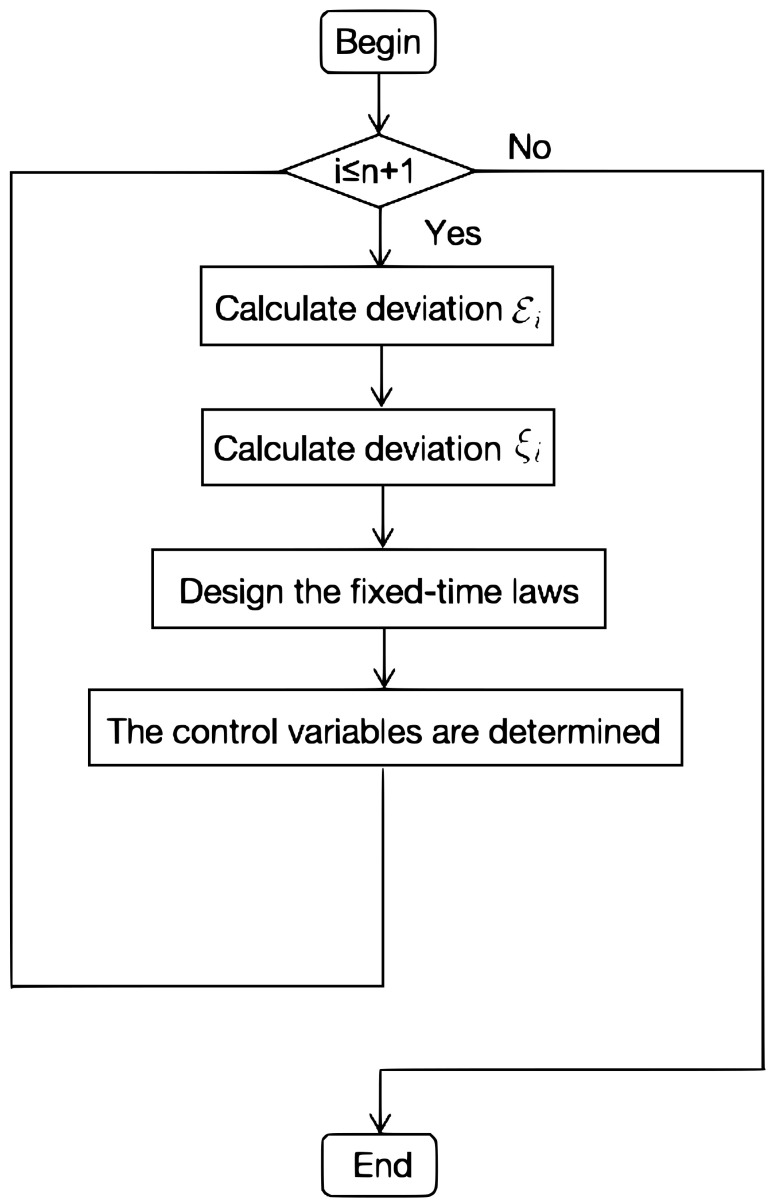
The fixed-time control algorithm.

The flow chart of the fixed-time control algorithm in this section is shown in Figure 1 below.

## 4. Simulation

**Figure 2:**
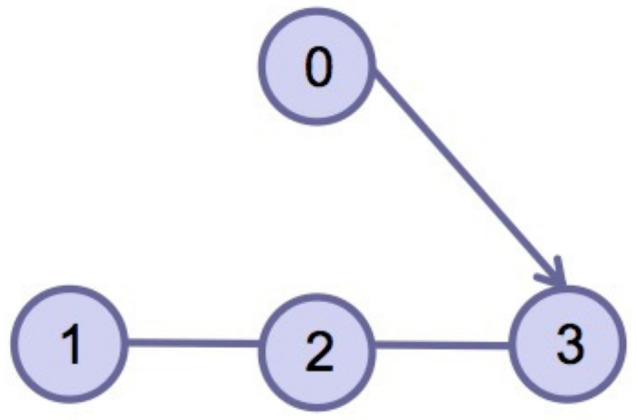
Subgraph $G_{\sigma (t)}^{1}$ in sub-interval $\left[t_{i}^{0},t_{i}^{1}\right)$.

**Figure 3:**
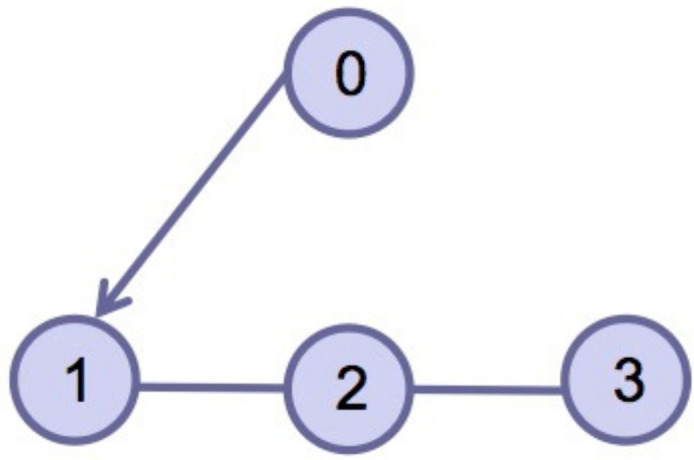
Subgraph $G_{\sigma (t)}^{2}$ in sub-interval $\left[t_{i}^{1},t_{i}^{2}\right)$.

**Figure 4:**
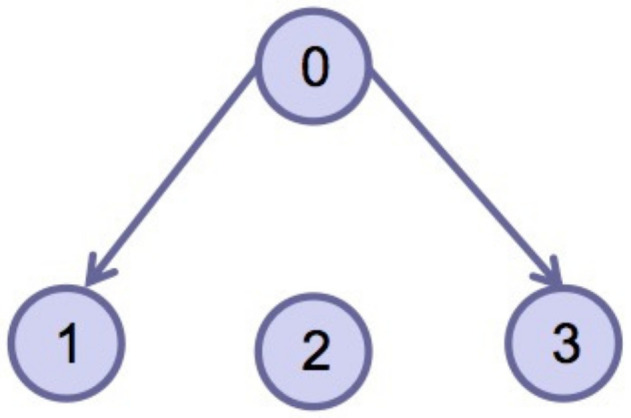
Subgraph $G_{\sigma (t)}^{3}$ in sub-interval $\left[t_{i}^{2},t_{i}^{3}\right)$.

**Figure 5:**
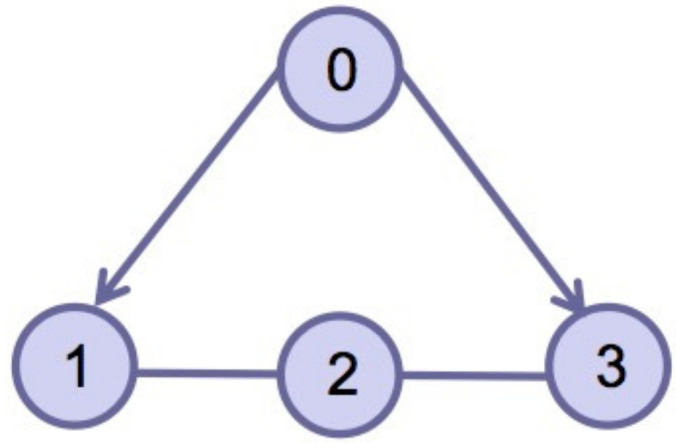
The jointly connected graph $G_{\sigma (t)}$ in time interval $\left[t_{i},t_{i+1}\right)$.

This section verifies the validity of the theoretical results through a simulation example. Consider four agents, one of which acts as the leader and is numbered 0, and the other three act as followers and are numbered 1–3. The dynamics of the four agents is shown in (2) and (3). Choose interval $\left[t_{i},t_{i+1}\right)$ and $p_{i}=3$; namely, interval $\left[t_{i},t_{i+1}\right)$ is divided into three sub-intervals $\left[t_{i}^{0},t_{i}^{1}\right)$, $\left[t_{i}^{1},t_{i}^{2}\right)$, $\left[t_{i}^{2},t_{i}^{3}\right)$, $t_{i}^{0}=t_{i}$, $t_{i}^{3}=t_{i+1}$, the subgraph of each sub-time interval is shown in Figure 2, Figure 3 and Figure 4. The jointly connected graph of the three subgraphs is shown in Figure 5.

Furthermore, the initial value of the leader is $x_{0}=9$, and the initial value of the followers is $x_{i}={\left[7,8,10\right]}^{T}$, while the adjacency matrix $A^{\sigma (t)}$ and the Laplacian matrix $L^{\sigma (t)}$ associated with Figure 5 are shown in (29) and (30). In addition, the nonlinear term of the leader is described by $f\left(t,x_{0}\right)=sin\left(x_{0}\right)$, and the nonlinear terms of followers are described by $f\left(t,x_{1}\right)=0.1sin\left(x_{1}\right)$, $f\left(t,x_{2}\right)=0.2sin\left(x_{2}\right)$ and $f\left(t,x_{3}\right)=0.3sin\left(x_{3}\right)$, respectively. Uncertain disturbances are regarded as $d_{1}\left(t\right)=sin\left(t\right)$, $d_{2}\left(t\right)=2sin\left(t\right)$ and $d_{3}\left(t\right)=3sin\left(t\right)$, respectively. Finally, let α=0.6, β=0.8, b=0.44 and γ=3.

(29) $$\begin{array}{c} A^{\sigma (t)}=\left[\begin{array}{cccc} 0 & 0 & 0 & 0\\ 1 & 0 & 1 & 0\\ 0 & 1 & 0 & 1\\ 1 & 0 & 1 & 0 \end{array}\right]. \end{array}$$

(30) $$\begin{array}{c} L^{\sigma (t)}=\left[\begin{array}{cccc} 0 & 0 & 0 & 0\\ -1 & 2 & -1 & 0\\ 0 & -1 & 2 & -1\\ -1 & 0 & -1 & 2 \end{array}\right]. \end{array}$$

**Figure 6:**
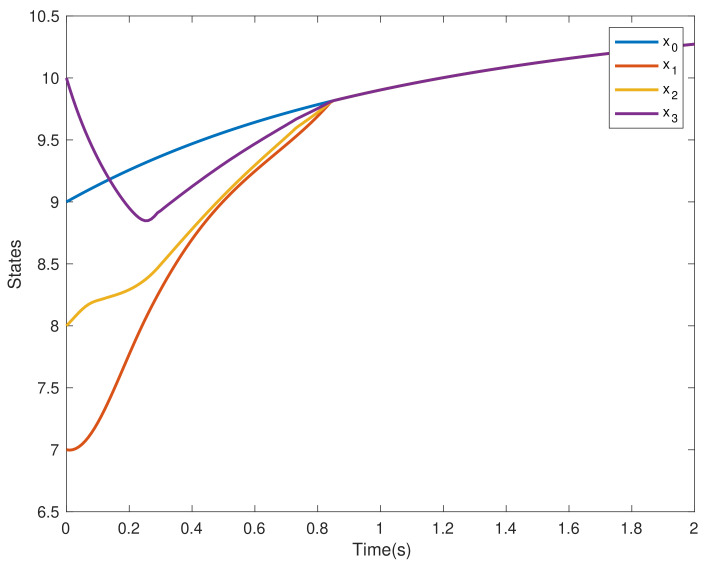
The states trajectories of the agents $x_{0},x_{1},x_{2},x_{3}$.

**Figure 7:**
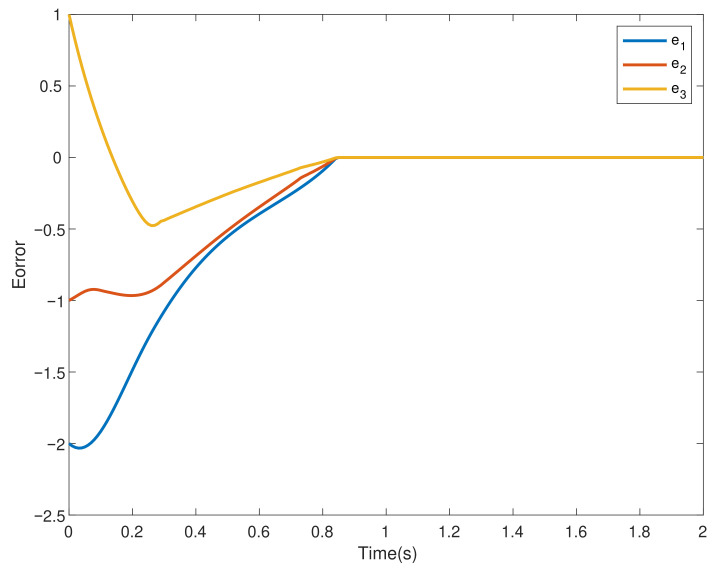
Trajectories of consensus errors $e_{i}$.

**Figure 8:**
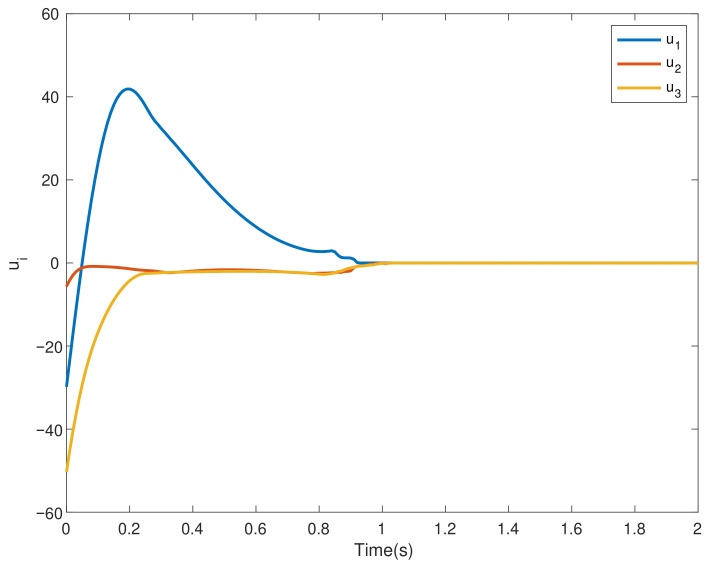
The control inputs $u_{i}$.

In the multi-agent systems composed of four agents, under the control (4), the states of the followers successfully converge to that of the leader agent within a fixed time independent of the initial value, as shown in Figure 6. The trajectories of consensus errors $e_{i}$ and the control inputs of each followers $u_{i}$ are given by Figure 7 and Figure 8, respectively.

## 5. Conclusions

In this paper, we research how to achieve fixed-time leader-following consensus for nonlinear multi-agent systems under a jointly connected graph. In addition, the system is affected by unknown disturbances. Compared with other studies in the literature on the fixed-time consensus problem, the advantage of this paper is that the unknown nonlinearity and unknown disturbances in the multi-agent systems can be solved under the jointly connected graph, simultaneously. Finally, this paper uses Matlab to carry out numerical simulation, which provides with a more intuitive proof of the theoretical part. In the future, the fixed time consensus problem of high-order nonlinear multi-agent systems can be solved.

## Figures and Tables

**Table 1:** Main notations table.

Notations	
R	The real number set
$R_{+}$	The positive real number set
$R^{n\times n}$	*n*-dimensional real vector space
1	The column vector of n×1 with all elements 1
$I_{n}$	The *n*-dimensional identity matrix
sign(·)	The sign function
$\lambda_{min}\left(\cdot \right)$	The smallest eigenvalue of the matrix
$\lambda_{max}\left(\cdot \right)$	The largest eigenvalue of the matrix

## Data Availability

Not applicable.
